# Uptake of 3‐iodothyronamine hormone analogs inhibits the growth and viability of cancer cells

**DOI:** 10.1002/2211-5463.12205

**Published:** 2017-03-06

**Authors:** Michael Rogowski, Lauren Gollahon, Grazia Chellini, Fariba M. Assadi‐Porter

**Affiliations:** ^1^ Department of Nutritional SciencesTexas Tech UniversityLubbockTXUSA; ^2^ Department of Biological SciencesTexas Tech UniversityLubbockTXUSA; ^3^ Department of BiochemistryUniversity of Wisconsin‐MadisonWIUSA; ^4^ Department of ZoologyUniversity of Wisconsin‐MadisonWIUSA; ^5^ Magnetic Resonance Facility at MadisonWIUSA

**Keywords:** 3‐Iodothyronamine, 3‐Iodothyronamine synthetic analog, cancer, metabolic substrate utilization, SG‐2, T1AM

## Abstract

3‐Iodothyronamine (T1AM) is a structural analog of thyroid hormone that has been demonstrated to have potent affects on numerous physiological systems. Most studies on T1AM have explored its effects in healthy functional systems; while its potential therapeutic uses and safety, and efficacy in pathological conditions are largely unknown. We sought to evaluate the effects of T1AM and its structural analog SG‐2 on cancer cell growth and viability. We analyzed the cytotoxicity of these analogs on MCF7 breast cancer cells, HepG2 hepatocellular cancer cells as well as normal control cells using primary human foreskin fibroblasts and mouse preadipocytes control cells. The cytotoxicity of T1AM and SG‐2 was determined by cell growth curves, and validated by 3‐(4,5‐dimethylthiazol‐2‐yl)‐2,5‐diphenyltetrazolium bromide cell viability assays. Cellular uptake analysis was conducted using confocal microscopy. Real‐time (RT)‐PCR was conducted to identify gene pathways affected by SG‐2 in cancer cells. The IC_50_ of T1AM was approximately double the concentration of its analog SG‐2 in cancer cells. Cytotoxicity studies on normal cells revealed that IC_50_ concentrations of SG‐2 in cancer cells had no significant impact on cell viability in these cell types. Cell‐imaging experiments demonstrated rapid uptake and localization to the mitochondrial membrane. T1AM and SG‐2 are able to reduce cancer cell growth and viability. These findings support the potential for use of these compounds and related analogs for their antiproliferation properties in cancer cells.

**Abbreviations**
BCL‐2: B‐cell lymphoma 2
FACS: fluorescent‐activated cell sorting
FL‐T1AM: fluorescent‐labeled T1AM
G6PD: glucose‐6‐phosphate dehydrogenase
GDH: glucose dehydrogenase
HFF: human foreskin fibroblast
HiF1α: hypoxia‐inducible factor 1 alpha
IC_50_: inhibitory concentration 50
LDHa: lactate dehydrogenase subtype a
MTT: 3‐(4,5‐dimethylthiazol‐2‐yl)‐2,5‐diphenyltetrazolium bromide
NF‐KB: nuclear factor kappa‐light‐chain‐enhancer of activated B cells
p53: tumor protein 53
PDHa: pyruvate dehydrogenase alpha
Sirt1: sirtuin 1
Sirt4: sirtuin 4
Sirt5: sirtuin 5
Sirt6: sirtuin 6
T1AM: 3‐iodothyronamin
T4: thyroxadine

3‐Iodothyronamine (T1AM) is a hormone‐like molecule that has recently been demonstrated to affect a wide variety of physiological systems 1, 2, 3. Because T1AM displays remarkable homology with thyroxadine (T4) hormone, it was initially proposed to be a product of deiodination of T4 pathways 4. Recent data suggest its synthesis is independent of T4 production, leaving its specific origins within the body unknown 5. Originally discovered as an activator of trace amine‐associated receptor 1 (TAAR1, a membrane spanning G protein‐coupled receptor), T1AM was able to induce dramatic decreases in core temperature, heart rate, and cardiac output in mice 1. The specific mechanisms of T1AM action in generating its metabolic effects currently remain unknown. However, some insights into its cellular signaling mechanisms suggest its effects are likely mediated through phosphorylation status of key cellular signaling molecules. It has been shown that T1AM's cardiovascular effects were potentiated by treatment with tyrosine kinase and phospholipase C inhibition, while being blunted with tyrosine phosphatase inhibition 6, 7. One striking physiological effect associated with acute high‐dose T1AM administration is the hypothermic effect shown to be maintained in TAAR1 knockout mice 8.

More recent research has demonstrated a promising application for T1AM to potentially treat obesity, causing weight loss by shifting macronutrient metabolism away from carbohydrates towards oxidation of fatty acids 9, 10. These shifts in macronutrient substrate utilization are supported by recent findings that chronic T1AM administration induces transcriptional activation changes consistent with a more lipolytic metabolic pattern 11. This shift moves toward a more metabolically healthy state in the obese condition as excess lipid is mobilized for energy.

The function of T1AM and how it reacts with cancer cells, as well as other pathological disease states, has not yet been explored in the literature. Cancer cell metabolism is unique, in that it is directed toward fueling rapid growth and division while repressing apoptosis. Fundamental to the process of fueling the rapid growth in cancer cells is the Warburg effect 12, whereby cancer cells consume large amounts of glucose, not only to fuel their metabolic needs but also for supplying the carbon substrates and reducing the factors needed for growth. Due to our laboratory's previous observations of metabolic shifting and weight loss in mice using T1AM 9, we hypothesized that T1AM may be able to disrupt cancer growth and proliferation, but at the same time sought to evaluate the cytotoxicity of the compound in normal cells, since the toxicity of T1AM has only been estimated *in vivo* based on low animal numbers 1.

Additionally, due to its therapeutic potential in a variety of physiological systems, functional analogs of T1AM have begun to be developed 13 (Fig. 1) with the goal of increasing its bioactivity. To this end, we sought to examine the potency of the synthetic T1AM analog, designated SG‐2, to evaluate enhanced potency compared to the native compound 15. The applications of T1AM and its analogs toward cancer treatment have not been previously explored. Here, we evaluate the impact of T1AM and SG‐2 on cancer cell growth rate *in vitro,* as well as their uptake patterns and gene transcription changes, in order to characterize their affects on cancer cells as well as their cytotoxicity in normal cells to determine their potential use in cancer therapy.

**Figure 1:**
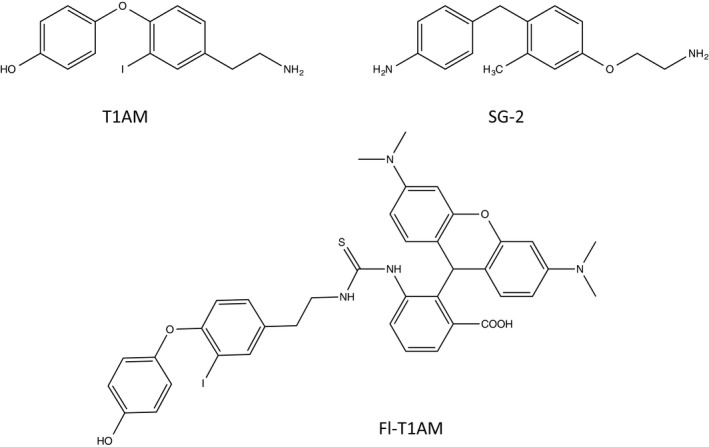
Structures of T1AM, SG‐2, and FL‐T1AM.

## Materials and methods

### Reagents and preparation of T1AM and SG‐2 stocks

Purified crystalline T1AM and SG‐2 (Fig. 1) were prepared as previously described 1, 14. Lyophilized powdered samples were first solubilized in DMSO before being diluting to a stock concentration of 2 mm using complete growth medium [10% FBS, 1% P/S, Dulbecco's modified Eagle's medium (DMEM)]. The hydrophobic nature of the two compounds necessitated the use of DMSO to enhance solubility in the aqueous solution. A small amount of DMSO was used to aid in the initial solubility of the compounds prior to the addition of media solution in order to aid its solubility in an aqueous solution. Stock solutions of compounds were prepared in batches of 1 mL by using 20 μL of DMSO to solubilize the compounds prior to adding the remaining volume of media. This created a stock solution containing 2% DMSO. This was done to keep the concentration of DMSO low so that even at the highest dosages (200 μm and above) the DMSO was only present at ~ 0.2% of treatment media. All control stock solutions had the equivalent amount of DMSO added to culture media so that the effects of DMSO would be present in controls. The addition of control media containing DMSO was added in amounts to reflect the highest dosage of compound with DMSO added to each series of experiments so that the potential effect of DMSO on cell growth was controlled for. Negative controls did not have any additional DMSO to their culture media. The authors do not believe this amount of DMSO had a significant detriment on cell proliferation of cells as no experiments indicated control growth rates were significantly less than negative controls.

### Measuring cell viability

Toxicity of T1AM and SG‐2 was assessed in MCF7 human breast adenocarcinoma cells, HepG2 heptocellular carcinoma cells, human foreskin fibroblast (HFF) normal HFFs, and 3T3‐L1 normal mouse preadipocyte fibroblasts in order to evaluate anticancer properties and cytotoxicity to normal cells. MCF7 and HepG2 were chosen for evaluation based on their divergent tissue origins. Similarly, 3T3‐L1 and HFF cells were chosen for evaluation based on ‘normal’ cell characteristics and different tissue origins to determine toxicity in different ‘normal’ cell types. Cells were seeded at different densities in a 96‐well plate and grown for 72 h in their respective standard growth media (DMEM 10%, FBS, 1% P/S) for MCF7, 3T3‐L1, and HepG2, and Medium 106 supplemented with Low Serum Growth Supplement (Invitrogen, Waltham, MA, USA) for primary HFF cells. After 72 h, cell viability was analyzed using the 3‐(4,5‐dimethylthiazol‐2‐yl)‐2,5‐diphenyltetrazolium bromide (MTT) assay. MTT was added at a final concentration of 0.5 mg·mL^−1^ and incubated for 4–6 h. Following incubation in MTT, cells were lysed in 2× formazan solubility solution (acidified isopropanol with 1% Triton X 100) and measured on a Cytation 3 plate reader (BioTek, Winooski, VT, USA) at 570 nm absorption with baseline subtraction of 630 nm. Viability data were then used to calculate the inhibitory concentration 50% (IC_50_). The lowest cell number at seeding that still produced peak MTT absorbance was utilized as the optimal plating density for future treatment experiments. Based on the growth rate or doubling time of each cell line, the optimal plating densities were as follows: MCF7 – 5000/well, HepG2 – 30 000/well, 3T3‐L1 – 7000/well, and HFF – 30 000/well. Each experiment was performed in replicates of six.

### Cellular toxicity

Cells were seeded according to their optimal 72‐h density in 96‐well plates and treated with multiple concentrations of SG‐2 or T1AM with vehicle (1% DMSO in serum‐free DMEM), or untreated. Treatment concentration ranges of T1AM and SG‐2 varied between cell lines and compounds in order to account for each cell line's respective tolerance levels. Cell viability was measured by MTT. The cell concentration that produced an MTT absorbance of 50% of the control treatment was designated the IC_50_ value. Cell morphological changes due to treatment were visualized utilizing an EVOS XL Core (Life Technologies, Carlsbad, CA, USA) digital imaging light microscope.

### Statistics

Significant differences in viability across T1AM or SG‐2 treatment concentrations were determined using one‐way ANOVA. Pairwise comparisons were performed using Dunnette's *post hoc* test compared to control. Dunnette's test was used because we were interested in differences in cell viability from the control rather than differences between incremental treatment dosages. Variability curves and IC_50_ doses were calculated using PROC REG in sas 9.4 (SAS Institute, Cary, NC, USA).

### Cell recovery growth

In order to determine if the decrease in growth rate due to SG‐2 treatment persisted past treatment cessation, cells were incubated over 7 days in growth media with or without SG‐2 at IC_50_ and were continually monitored for an additional 7 days post‐treatment. Cells were seeded into 48‐well plates. Cells from multiple wells per treatment (three to four wells per time point) were counted using a hemocytometer and Trypan blue exclusion staining for days 0, 1, 4, 7 of treatment and days +1, +4, +7 post‐treatment, following the removal of the drug from the growth media. Significant differences between treatments were statistically determined for each time point using an independent *t*‐test.

### Development of fluorescently labeled T1AM

The specific mechanism of T1AM entry into the cell, as well as its internal target or areas of compartmentalization, remains unknown. To address this, a fluoro‐labeled version of T1AM (FL‐T1AM) was synthesized by conjugating T1AM to rhodamine (Fig. 1), previously shown to be effective in creating fluoro‐conjugates of thyroid hormone derivatives 15.

### Intracellular localization of T1AM

Cells were plated onto 35‐mm glass bottom confocal imaging plates (MatTek, Ashland, MA, USA) at 100 000–250 000 cells per plate, 24 h prior to imaging. To image the FL‐T1AM at 561/610 nm excitation/emission, cells were incubated with a combination of dyes for 20–40 min in serum‐free media then washed. The combination of cell dyes used in each experiment varied depending on the specific cell regions imaged, and were selected to avoid spectral overlap. Dyes included: CellMask™ Green Plasma Membrane Stain excitation/emission 522/535 nm at 5 ng·mL^−1^, (Invitrogen), MitoTracker® Deep Red FM excitation/emission ~ 644/665 nm at 150 nm (Invitrogen), and DAPI nuclear stain excitation/emission 358/461 at 1 μg·mL^−1^ (Invitrogen). Confocal images were taken using an Olympus IX81 inverted microscope (Olympus Corporation, Tokyo, Japan) with a Yokagawa X1 Spinning Disk confocal box. Laser launch contains four lasers, 405 nm, 488 nm, 561 nm, and 647 nm. The images were captured at 60× using a Photometrics Evolve 512 EMCCD camera and images were analyzed in Slidebook (3i Intelligent Imaging Innovations, Denver, CO, USA). Once baseline cell images were taken, FL‐T1AM was added to media at 10 nm and imaged over real‐time to assess T1AM uptake and cellular localization.

### Cellular uptake kinetics

In order to assess the rate and pattern of T1AM uptake into the cells, the FL‐T1AM conjugate was used to track its emission signal in cells over time by flow cytometry. Measurements were conducted on a BD fluorescent‐activated cell sorting (FACS) Aria III (BD Bioscience, San Jose, CA, USA). Each data acquisition was 10 000 cells over 30 s with 1 min between readings over the course of 15–20 min depending upon uptake plateau and available sample. ~ 1 million cells in 1 mL kept on ice were sampled, prior to the addition of FL‐T1AM, to establish baseline readings. FL‐T1AM was added at 10 nm to the cell media and immediately measured for emission signal detection and measured repeatedly to assess shift in signal detection over time at room temperature. This was conducted in MCF7 cells pretreated for 24 h with control growth media as well as media supplemented with 27.5 or 55 μm of SG‐2 to assess the impact of SG‐2 on T1AM uptake.

### Gene expression study

Metabolic genes were profiled by RT‐PCR as previously described 16. Primers were designed in‐house using beacon designer Software v.7.9 (Premier Biosoft International, Palo Alto, CA, USA) with a junction primer strategy (Table S1). Total RNA was isolated with RNeasy Lipid Tissue Mini kit (Qiagen GmbH, Hilden, Germany). RNA integrity, concentration, and purity were evaluated using Experion (Bio‐Rad Laboratories, Hercules, CA, USA), and Cytation (Cytation 3, Winooski, VT, USA), respectively. RNA was retrotranscribed using iScript cDNA Synthesis Kit (Bio‐Rad Laboratories). Relative quantification of gene transcripts was quantified using SYBRGreen by RT‐PCR (Bio‐Rad Laboratories). For data normalization, reference gene β‐actin was used. All reactions were run in duplicates. Results were analyzed by the 2‐ΔΔCt method as described 17.

### ^1^H‐NMR metabolomics

Cells were collected in 20 mm sodium phosphate buffer and lysed by repeated freeze–thaw cycles prior to being placed in a speed vacuum to dry overnight. The dried supernatant was redissolved in D_2_O containing 1 mm 4,4‐dimethyl‐4‐silapentane‐1‐sulfonic acid, and 0.1 mm sodium fluoride and the pH was adjusted to 7.4 ± 0.05. One‐dimensional (1D) spectra of glucose and lactic acid were collected on at 25°C on a 600 MHz Varian (Palo Alto, CA, USA) nuclear magnetic spectrometer equipped with a cryogenic probe 18. Each 1D spectrum was accumulated for 1028 scans, with an acquisition time of 400 ms (4096 complex points) and a 3‐s repetition delay for a total collection time of ~ 2 h. 1D data were then analyzed using Chenomx NMR suite (Chenomx, Edmonton, Alberta, Canada) software to identify relative concentrations of metabolites of interest.

## Results

### Evaluation of cytotoxicity for T1AM and SG‐2

**Figure 2:**
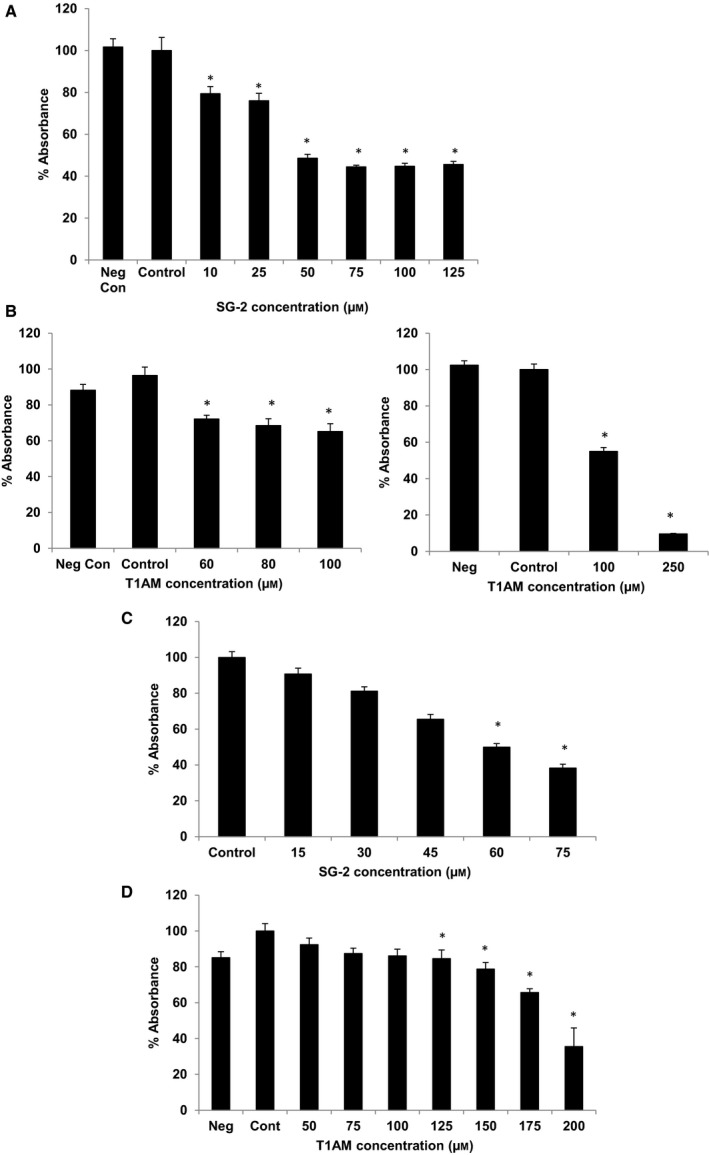
Growth inhibition in cancer cells due to SG‐2 and T1AM treatment. SG‐2 and T1AM inhibit cancer cell growth. *Y*‐axis displays % absorbance of control treatment with control set at 100%. *X*‐axis is μm of SG‐2 or T1AM with control representing 0 μm with treatment vehicle (Cont) and negative control (Neg) indicating untreated. SG‐2 appears to be twice as potent as T1AM at inhibiting cancer cell growth with an IC_50_ of 55 μm in MCF7 and 62 μm in HepG2. In contrast, T1AM estimated IC_50_ in MCF7 was 120 μm. (A) Decreased cell viability in response to SG‐2 and (B) T1AM incubation over 72 h on MCF7 cells across increasing concentrations. (C) Decreased cell viability in response to SG‐2 and (D) T1AM incubation over 72 h on HepG2 cells across increasing concentrations. * indicates significant difference (*P* < 0.05) from control.

**Figure 3:**
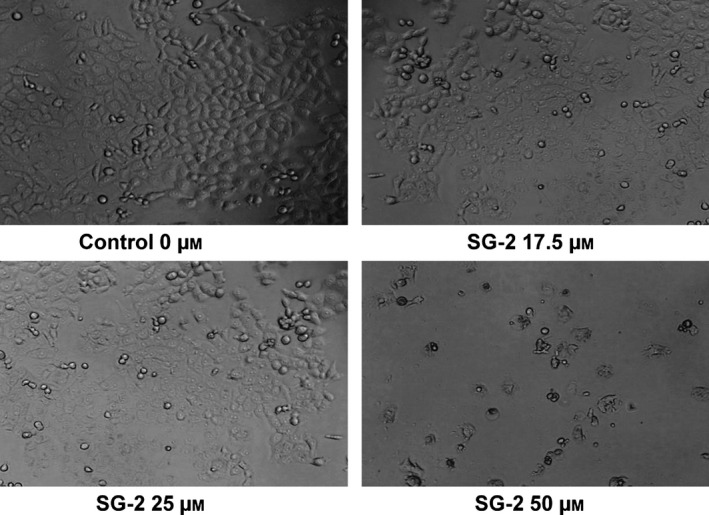
Morphological changes due to SG‐2 treatment in cancer cells. Treatment of cancer cells with SG‐2 causes morphological changes indicative of cell stress and membrane disruption consistent with decreased cell viability. Representative fields of view of MC7 cells treated with various dosages with SG‐2 over 72 h. Cells were seeded at equal cell density of 5000 cells per well on a 96‐well plate. Cells were visualized under 20× magnification.

Our results showed that incubation of MCF7 cells with T1AM and SG‐2 over 72 h resulted in significant decrease in cell viability for both compounds (Fig. 2A,B). The IC_50_ for T1AM and SG‐2 was 88 μm and 56 μm, respectively (Table 1). Treatment of HepG2 cells demonstrated a similar response to T1AM and SG‐2 exposure (Fig. 2C,D) suggesting their potential for antiproliferative effects across a variety of cancer types. The IC_50_ value of T1AM was 2.6× higher than SG‐2 (158 μm compared to 60 μm) in HepG2 cells compared to 1.6× higher than SG‐2 in MCF7 (Table 1), which may reflect differences between the cell linage and growth rates of the two cell lines and the potency of the compounds. While informative, the MTT assay does not distinguish between a decline in metabolic function and a reduction in cell number. Thus, cells were visually assessed and photographed after 72 h incubation with SG‐2 (Fig. 3) revealing irregular, more rounded cells, indicative of cell stress and/or cell death at the higher concentrations of SG‐2 in the media.

**Table 1:** IC50 of T1AM and SG‐2 on cell lines

Cell line	Treatment	IC_50_ (μm)
MCF7	T1AM	88
	SG‐2	56
3T3‐L1*	T1AM	112
	SG‐2	–
HepG2	T1AM	158
	SG‐2	60
HFF	T1AM	176
	SG‐2	173

*IC50 of 3T3‐L1 was not able to be calculated as treatment values did not approach close enough to 50% to provide an accurate estimate.

**Figure 4:**
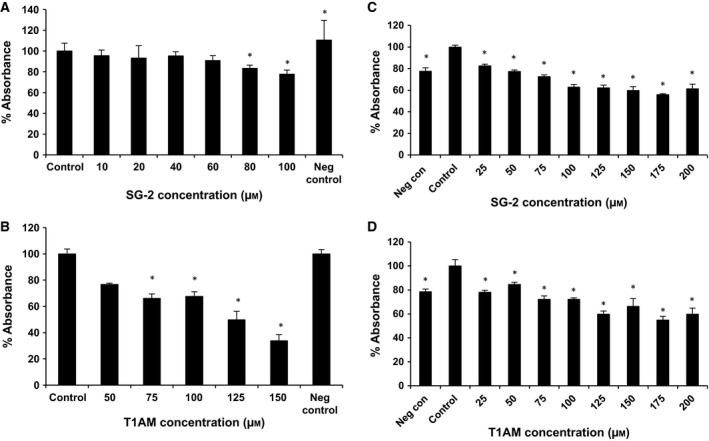
Cytotoxicity effects of SG‐2 and T1AM treatments in normal cells and cancer cells. *Y*‐axis displays % viability of control treatment with control treatment set at 100%. *X*‐axis is μm of SG‐2 or T1AM with control representing 0 μm with treatment vehicle and untreated control (Neg. Con). SG‐2 and T1AM have minimal impact on cell viability in normal cells even at higher μm concentrations as opposed to cancer cells. (A) Impact on cell viability in response to SG‐2 in 3T3‐L1 cells. (B) Impact on cell viability in response to T1AM in 3T3‐L1 cells. (C) Impact on cell viability in response to SG‐2 in HFF cells. (D) Impact on cell viability in response to T1AM in HFF cells.* indicates significant difference (*P* < 0.05) from control viability. Error bars indicate ± SEM.

**Figure 5:**
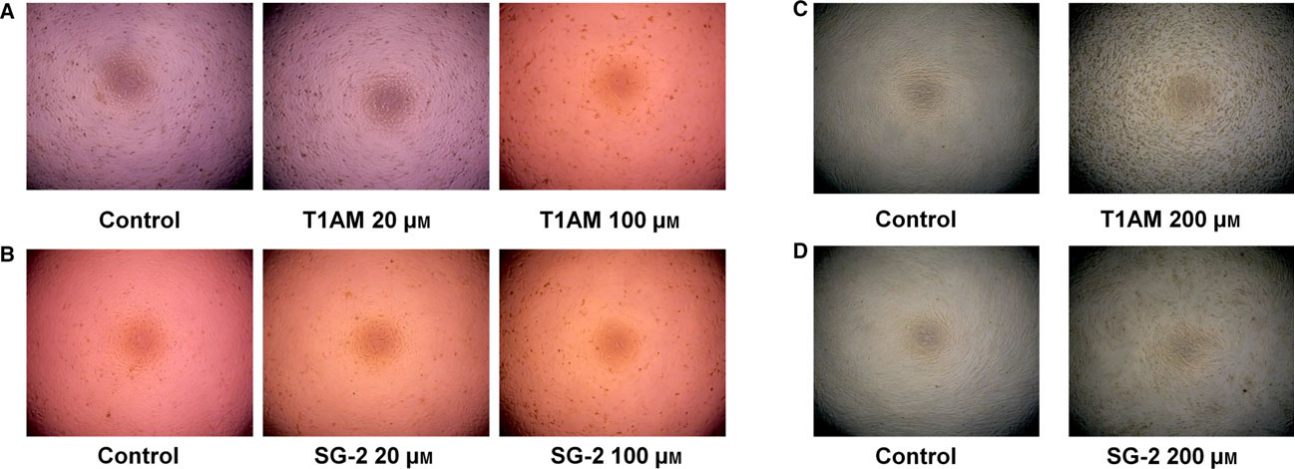
Growth of normal cells treated with T1AM and SG‐2. Treatment of normal cells with T1AM or SG‐2 did not induce adverse morphological changes comparable to those seen in cancer cells until much higher dosages. Representative fields of view of 3T3‐L1 or HFF cells treated with various dosages of T1AM or SG‐2 over 72 h. Cells were seeded at equal cell density within cell type per well on a 96‐well plate. Cells were visualized under 20× magnification. (A) Growth of 3T3‐L1 cells T1AM treated. (B) Growth of 3T3‐L1 cells SG‐2 treated. (C) Growth of HFF cells T1AM treated. (D) Growth of HFF cells SG‐2 treated.

The potential cytotoxicity to normal cells was evaluated using cell lines from two different tissue types; the mouse 3T3‐L1 pre‐adipocyte and HFF cells (Fig. 4). In 3T3‐L1 cells, SG‐2 did not significantly impact cell viability until 80 μm, hence an IC_50_ value was not able to be determined as the observed percent drop in viability did not approach 50% (Table 1). The concentration required to negatively affect cell viability in 3T3‐L1 was considerably higher than that of MCF7 cells (Fig. 2), suggesting SG‐2 is even less cytotoxic in normal cells. The IC_50_ of T1AM in 3T3‐L1 was 112 μm, which was not a particularly far range from that of MCF7 cells (88 μm). These data suggest that SG‐2 is more potent against cancer cells as well as less harmful to normal cells than its native analog. In the HFF cells, viability was comparable between SG‐2 and T1AM (173 and 176 μm, respectively) with very modest incremental drops in viability compared to control at dosages up to 200 μm. This is collaborated with gross visual observations of cell morphology in both 3T3‐L1 cells and HFF (Fig. 5) that were not visually apparent until 200 μm. Confluency and cell morphology were largely maintained in 3T3‐L1 cells up to 100 μm. HFF cells did not display noticeable changes in morphology until 200 μm in either treatment, with 200 μm of SG‐2 resembling the control cells much more so than T1AM 200 μm (Fig. 5C,D).

### SG‐2 treatment attenuates cancer cell proliferation

**Figure 6:**
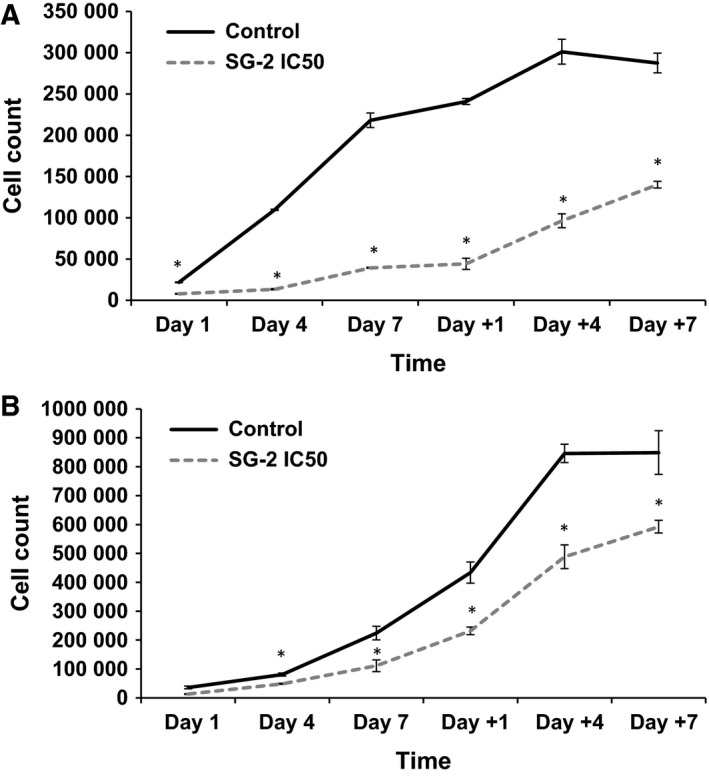
Recovery of cancer cells from SG‐2 treatment. SG‐2 treatment continues to inhibit cell growth rate even after removal of treatment. *Y*‐axis indicates average cell count per well over days of treatment (Day 1, Day 4, Day 7), and days of recovery after treatment (Day +1, Day +4, Day +7). (A) Recovery of MCF7 cells from IC_50_ concentration of SG‐2. (B) Recovery of HepG2 cells from IC_50_ concentration of SG‐2. * indicates significant difference between control and treatment (*P* < 0.05). Error bars indicate ± SEM.

After establishing the efficacy of SG‐2 on inhibiting cancer cell growth during treatment, we investigated whether there was a more long‐term impact on cell proliferation upon removal. Many drug therapies are only effective in the presence of the drug, but due to side effects or tolerance issues, long‐term continuous usage is not always advocated. To that end, using a recovery protocol we evaluated whether SG‐2 treatment effects were prolonged in cancer cells even after removal (Fig. 6). Results showed that growth rates of cancer cells continue to be suppressed compared to untreated controls and did not reach comparable numbers even after 7 days of recovery. This suggests that SG‐2 treatment had a sustained negative effect on cancer cells proliferation, up to a week after treatment cessation.

### The intracellular fate of T1AM

**Figure 7:**
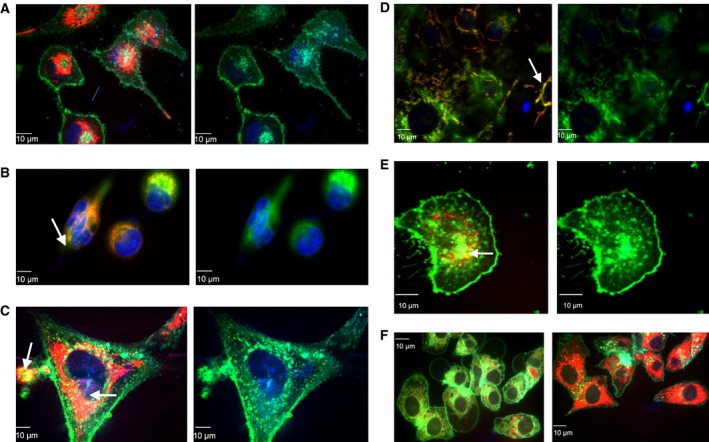
Cellular localization of T1AM. T1AM is rapidly taken up into cells, localizing to mitochondria while remaining perinuclear in its intracellular distribution. (A) MCF7 cells with FL‐T1AM (left) and without FL‐T1AM (right) signal. Cells were counterstained with cell membrane dye. (B) MCF7 with FL‐T1AM (left) and without FL‐T1AM signal (right). Cells were counterstained with mitochondria membrane stain. (C) 3T3‐L1 cells with FL‐T1AM (left) and without FL‐T1AM signal (right). Cells were counterstained with cell membrane stain. (D) 3T3‐L1 cells with FL‐T1AM (left) and without FL‐T1AM signal (right). Cells were counterstained with mitochondrial membrane dye. (E) MCF7 cells incubated with FL‐T1AM (left) and without FL‐T1AM signal (right). Cells were incubated with IC_50_ of SG‐2 overnight prior to imaging and counterstained with cell membrane stain. (F). Changes over time in FL‐T1AM signal in HepG2 cells. Left is FL‐T1AM signal at 10 s and right is after 20 min of incubation with FL‐T1AM. For all images Blue = DAPI nuclear stain; red = FL‐T1AM; green = cell membrane stain for images A,C,E, and F, and mitochondria membrane stain for images B and D; yellow and orange signal indicates colocalization of red and green signals between FL‐T1AM and mitochondria membrane see: arrows in images B and D.

While T1AM has been studied for over 10 years 1 its mechanisms of cellular uptake, action and cellular targets remain unclear. To gain insight into its pattern of cellular uptake, a T1AM‐fluorescent conjugate was used to track its intracellular fate. Historically, T1AM had been thought to primarily work through cell surface receptors, and previous studies provided indirect evidence of cellular internalization 19, 20. Our tracking results confirmed that T1AM was taken up by the cells (Fig. 7). However, an unexpected observation was the extremely rapid rate of uptake into the cell during imaging (< 10 s) with a subsequent increase in signal intensity over time (Fig. 7F). This observed rapid uptake supports previous studies showing T1AM's intracellular uptake involves facilitated diffusion mechanisms 21. FL‐T1AM signal appeared as clustered aggregates, suggesting vesicular compartmentalization as it moved from the outer edges of the cell inward (Fig. 7A,C). Importantly, the FL‐T1AM signal overlapped with mitochondrial membrane signal, suggesting mitochondrial colocalization (Fig. 7B,D).

**Figure 8:**
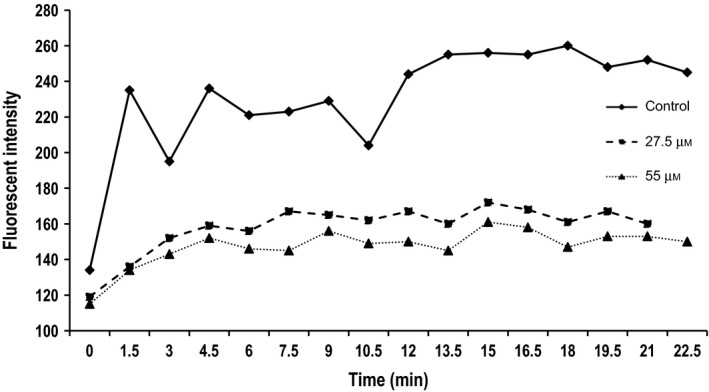
Competitive inhibition of cellular FL‐T1AM uptake kinetics in cancer cells pretreated with SG‐2. MCF7 cancer cells (10^6^) were incubated with 10 nm of FL‐T1AM. Each measurement counted 10 000 cells. MCF7 cells were pretreated overnight with SG‐2 at 0 (Control, solid line), half of IC_50_ (27.5 μm, dashed line) and IC_50_ (55 μm, dotted line) concentrations prior to flow cytometry. *Y*‐axis indicates mean fluorescent intensity (arbitrary units) and *X*‐axis is in time in min. FL‐T1AM uptake rate and signal plateau indicated competitive inhibition in cells pretreated with SG‐2, suggesting similar uptake mechanisms for T1AM and SG‐2 with saturatable uptake rates.

The amount of FL‐T1AM absorbed by cells was concentration dependent to such a degree did not observe a limit to its increase uptake across a wide range of dosages (data not shown). Therefore, in order to determine if there were absorption limits and whether the mechanism and rate of cellular uptake was mirrored with SG‐2 treatment, cells were preincubated with SG‐2 for 24 h prior to imaging with FL‐T1AM. The results showed that compared to controls with no pretreatment, FL‐T1AM uptake was slower in preloaded MCF7 cells. This suggested that T1AM's and SG‐2′s uptake mechanisms are similar, if not identical due to their homology, if SG‐2 uptake is able to interfere with FL‐T1AM uptake (Fig. 7E). In order to confirm this, the impact of SG‐2 treatment on T1AM uptake was quantitatively analyzed using FACS (Fig. 8). Based on the FACS data, FL‐T1AM uptake rate and signal plateau indicated competitive inhibition in cells pretreated with SG‐2, suggesting similar saturated uptake rate mechanisms for T1AM and SG‐2.

### Gene expression changes in MCF7 cells post‐SG‐2 treatment

**Figure 9:**
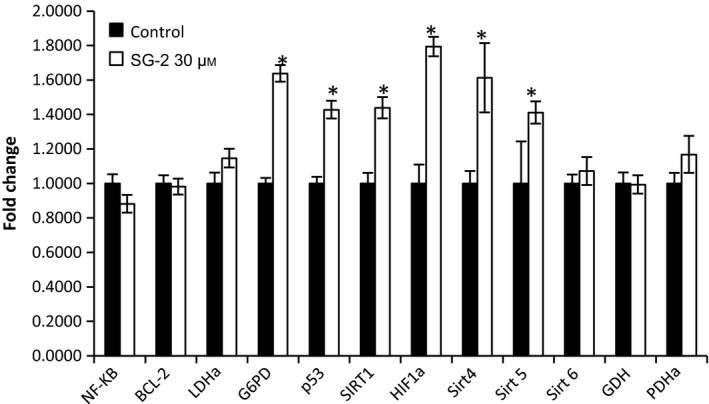
SG‐2 treatment results in gene expression changes in MFC7 cells. MCF7 cells were treated with 30 μm SG‐2 for 24 h. Genes associated with apoptosis, glucose metabolism, and sirtuins were analyzed for changes due to SG‐2 treatment. Gene expression changes were analyzed for relative gene expression via real time quantitative PCR and the 2^∆∆^ ^CT^ method. Significant changes in gene expression were observed for Sirt1, 4, and 5, G6PD, p53, and HIF1α. * indicates significant difference between fold expression (*P* < 0.05). Error bars indicate ± SEM.

In order to begin elucidating pathways affected by SG‐2 in cancer cells, selected genes encompassing multiple metabolic processes, including fatty acid oxidative metabolism, tumorigenesis, and glycolytic metabolism, were analyzed for gene transcriptional changes. Interestingly, results showed increases in hypoxia inducible factor 1 alpha (HiF1α; a cellular redox‐sensor and a driver of mitophagy and autophagy), and glucose‐6‐phosphate dehydrogenase (G6PD) an important regulator of the pentose phosphate pathway and key supplier of a reducing agent in form of NADPH to cells), suggesting acute response to hypoxic or oxidative stress in the cancer cells. Increase in sirtuin gene transcription (Sirt 1, 4, and 5, post‐translational modifiers of metabolic and ketogenic enzymes) levels were also observed, in addition to increased expression of the antitumor protein p53 (Fig. 9). Possible implications for these results are discussed below.

### Metabolome profiling of cancer cells

**Figure 10:**
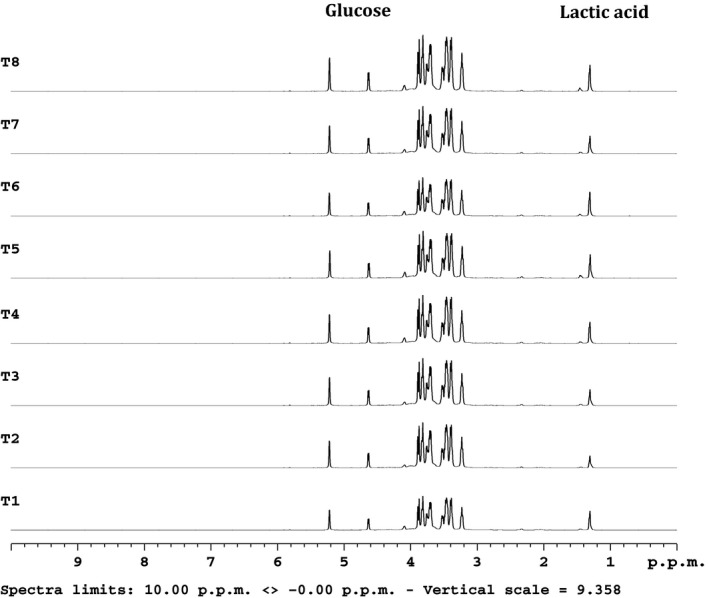
Metabolome profiling of HepG (carcinoma cells) by ^1^H NMR spectroscopy. T1AM treatments show lactate and glucose normalization over an 8‐h interval. Lactic acid and glucose are as labeled. Spectra are collected at 600 MHz Varian machine. Traces from bottom to top are time dependent of HepG cells in response to 1 μm T1AM from T1 to T4 corresponding to 1 h to 4 h, then treated with a higher dose (5 μm) of T1AM from T = 5–8 h. Data were collection for 10^6^ cells/experiment.

Due to our previous work in examining the metabolic substrate shift toward lipid utilization that T1AM can induce in an *in vivo* system 9 we initially hypothesized that T1AM and its analogs may be able to disrupt cancer cell metabolism by shifting substrate utilization away from glucose, disrupting the Warburg effect. HepG2 cancer cells were subjected to 1 μm for 1–4 h and further treated at 5 μm for 5–8 h. This T1AM treatment over the 8‐h interval resulted in lactate and glucose normalization (Fig. 10), supporting the likelihood of diminished glucose utilization as a mechanism of reduced growth and viability in cancer cells mediated by T1AM and SG‐2 treatment.

## Discussion

Our results showed that incubation of MCF7 cells with T1AM and SG‐2 over 72 h resulted in significant reduction in cell viability, IC_50_ 88 and 56 μm, respectively (Table 1). Furthermore, SG‐2 was also effective in reducing cell viability in HepG2 cells, a cancer cell line of dramatically different tissue origin and lineage (Fig. 2B). Cytotoxicity results in normal cells demonstrated that SG‐2 was far less toxic compared to similar doses in cancer cells. Indeed, cytotoxicity was not observed to significantly affect cell growth in 3T3‐L1 cells until a dosage 2× greater than the IC_50_ dosages in cancer cells (Fig. 4). Interestingly, a cell lineage‐specific response appears to play a role in cell sensitivity to treatment by T1AM and SG‐2. While 3T3‐L1 cells demonstrated decreases in viability at 75 μm (comparable to 60 μm in MCF7; Fig. 4), cell viability in HFF cells did not begin to approach an IC_50_ value with SG‐2 or T1AM treatments until concentrations of 175 μm. It is not yet clear from these initial experiments how T1AM and SG‐2 are impacting the viability of cancer cells, if it is mediated more through interruption of the cell cycle or inducing apoptosis, but it is observable that it results in substantial changes to cell morphology (Fig. 3) rather than mere cessation of proliferation. Furthermore, 3T3‐L1 cells possess a rapid doubling time comparable to MCF7s but do not suffer cell cycle arrest at equivalent dosages, suggesting that T1AM and SG‐2 may be inducing apoptosis in cancer cells.

Because many drug therapies are only effective when the drug is present, and the side effects or tolerance issues prevent long‐term, continuous treatment, we evaluated whether SG‐2 exposure had a prolonged effect on cancer cell growth, even after being removed from the media (Fig. 6). Results showed that not only were growth rates of cancer cells continuously inhibited compared to untreated controls but the cell population doubling time was also slower during the recovery period compared to the untreated cells (data not shown). These data coupled with the low cytotoxicity of SG‐2 suggests that utilizing this thyroid hormone analog in combination with other anticancer drugs with different mechanisms of action, such as doxorubicin, may yield better treatment results with less toxic dosages over a prolonged period of time.

Although T1AM had initially been thought to primarily work through surface receptors on the cell, previous studies provided indirect suggestions of cellular internalization 19, 20. Our results showed a surprisingly rapid cellular uptake of FL‐T1AM, which resulted in a signal that localized internally within seconds of its addition to the cell media. The ease by which T1AM entered the cells suggests that internalization may be even more important to its function than its surface receptor interactions, previously thought to be its primary pathway of cellular activation 1. Fluorescence microscopy elucidated several new insights involving cellular uptake of T1AM. When visualized in conjunction with a cellular membrane dye (Fig. 7A,C, and E), a distinct outline of circular clusters appears. This suggests that T1AM is rapidly taken up irrespective of cell type, and stored through vesicle formation and subsequent trafficking. The degree to which the FL‐T1AM is taken up is quite remarkable (Fig. 7F). Our observations involving the rapid uptake of FL‐T1AM appeared to confirm previous studies suggesting that T1AM entered the cell via a facilitated diffusion mechanism 21, since the low concentrations of FL‐T1AM (~ 10 nm) were enough to create a strong signal rapidly, observed in real‐time under confocal microscopy. At ~ 10 nm such a robust response produced so quickly seems unlikely without the involvement of facilitated diffusion.

We also wanted to evaluate if the mechanism of cellular uptake for SG‐2 and T1AM uptake were similar due to their homology. To address this, cells were pre‐incubated with SG‐2 over 24 h prior to imaging with FL‐T1AM (Fig. 7E). Interestingly, SG‐2 pretreatment interfered with FL‐T1AM uptake. The reduced uptake seen in MCF7 cells pretreated with SG‐2 supports this possibility (Fig. 8). This decreased uptake of FL‐T1AM observed due to SG‐2 treatment cannot be attributed to cell death either, as incubation with SG‐2 at IC_50_ and one half of the IC_50_ concentration showed similar patterns of decrease in FL‐T1AM uptake that is not in proportional to the potential in growth reduction for the respective dosages. This suggests that the homology of SG‐2 and T1AM is linked to uptake, whereby the relative excess of SG‐2 inside the cell prohibited further T1AM uptake into the cell.

One of the most important observations to emerge from fluorescent imaging analysis, however, is the FL‐T1AM colocalization to the mitochondrial membrane (Fig. 7B,D). These results provide direct evidence for the mitochondria as one of the primary targets of T1AM. Also notable is that the FL‐T1AM did not incorporate into the nucleus. This result is in stark contrast to the function of its related hormone molecules T4 and T3, which primarily exert their intracellular action through activation of THα and THβ receptor transcription factors in the nucleus 1. Although our data also demonstrate transcriptional changes in response to T1AM treatment, image analysis presented here demonstrates that T1AM does not interact directly with nuclear transcription factors. This result suggests that gene transcription may be mediated through indirect interactions via secondary messenger systems, or activation of transcription regulators that lie outside the nucleus and can be imported (e.g., Sirt1), or that the metabolic effects observed with T1AM administration 9 may be primarily mediated by the mitochondria directly rather than through the nuclear transcript activity as shown for thyroid hormone.

How T1AM and SG‐2 were exerting their anticancer effects was not readily apparent. In order to elucidate the pathways affected by SG‐2 in cancer cells, a number of genes spanning multiple metabolic processes to include fatty acid oxidative metabolism, tumorigenesis, and glycolytic metabolism were analyzed for transcriptional activation. Given our previous findings demonstrating an ability of T1AM to alter macronutrient metabolism 9 under normal conditions, we suspected in part that these decrements in cancer cells that spared normal cells may be related to alterations in cellular fuel metabolism, possibly disrupting the Warburg effect 12. However, the results for key genes analyzed, related to glucose metabolism showed no changes in levels of lactate dehydrogenase subtype a (LDHα), pyruvate dehydrogenase alpha (PDHα), or glucose dehydrogenase (GDH; Fig. 9), which indicates that this particular effect of T1AM and its analog SG‐2 may not be the primary means by which it suppresses cancer cell proliferation, at least at the transcriptional level. However, despite a lack of change in the transcription of these metabolic regulators, ^1^H‐NMR metabolomics data did indicate that T1AM decreased lactic acid and regulated glucose levels (Fig. 10), helping to normalize glucose metabolic flux in HepG2 cells over time. The fact that this effect is observed in cancer cells but not reflected in genomic transcription changes, combined with our cellular uptake data (Fig. 7), further supports the primary role of T1AM and SG‐2′s action in the mitochondria.

**Figure 11:**
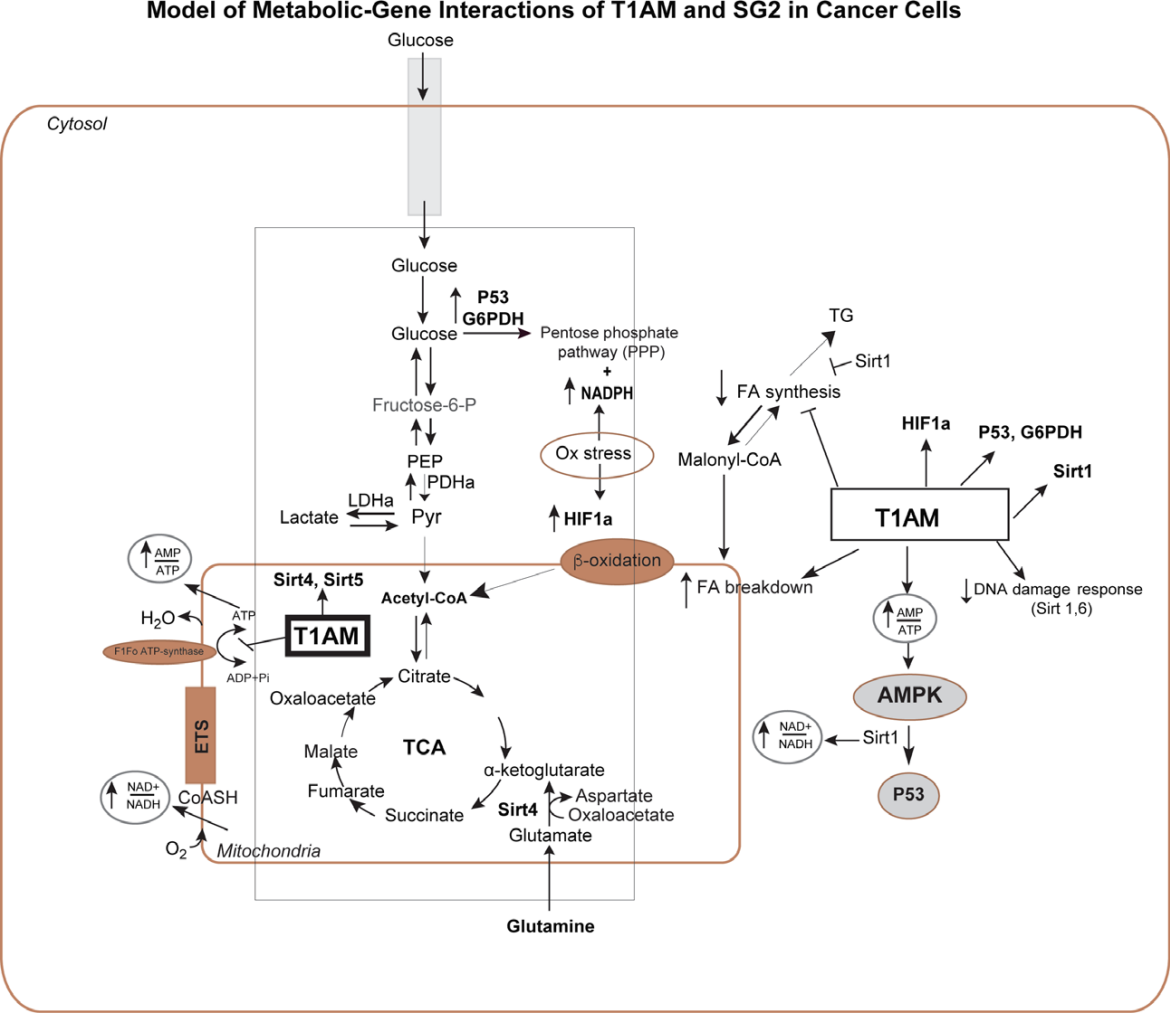
Proposed model for metabolic‐gene interactions of T1AM and SG‐2 in cancer cells. Our work indicates that T1AM localizes into mitochondria that coincides with upregulation in expression of mitochondrial genes, Sirt 4 and 5, by SG‐2 in cancer cells. This suggests that one possible mechanism for interaction of T1AM and SG‐2 in cancer cells is mitochondria driven and maybe through sirtuin‐mediated pathways. Additionally, this mechanism is consistent with previous research indicating that T1AM could inhibit F_0_F_1_‐ATP synthase of electron transport system at sufficiently high dosages 3. Other upregulation of genes such as G6PD, p53, HIF1α, and Sirt 1, which are localized in cytosol or nucleus maybe through indirect metabolic effects of these compounds in cells through yet additional unknown mechanism leading to observed normalization of glucose and lactic acid in cancer cells.

Further gene transcript paneling of genes related to cancer activity in cells also showed no changes with both nuclear factor kappa‐light‐chain‐enhancer of activated B cells (NF‐κβ), and B‐cell lymphoma 2 (BCL‐2) expression. In contrast, an upregulation in G6PD was observed, which we initially expected would be downregulated as G6PD is not only a crucial enzyme for the pentose phosphate pathway in the biosynthesis of fatty acids 22 (Fig. 11) but also a key source for generating NADPH as a reducing agent in cells and fueling other essential reducing agents such as glutathione. Interestingly we observed increased expression of multiple sirtuins: Sirt 1, 4, and 5. Sirtuins are a new family of cancer cell target proteins that are involved in post‐translational modifications in addition to cellular metabolism. Sirtuins play a key role as metabolic sensors in regulating fat and glucose metabolism in response to physiological changes in energy levels 23. Thus, they may provide a novel mechanism to regulate on/off action of several key metabolic enzymes in tumor cells.

Perhaps the most significant gene expression shift identified from these data may be from the increased levels of HiF1α, which is indicative of the cellular response to increased levels of environmental stress. Since the cells were grown under controlled culture conditions, this response is not likely to be due to a lack of oxygen. However, HiF1α has been shown to be upregulated in response to elevations of reactive oxygen species 24. Furthermore, upregulation of HiF1α is known to be a driver of both mitophagy and autophagy 25. This may be a potential mechanism for the cellular distress observed in cancer cells in response to SG‐2 treatment. How this particular gene expression pattern reflects the pathways by which cancer proliferation is altered is not clear, but the particular points at which these genes interact with cellular metabolism is, however, somewhat known, which may provide a basis for future studies in assessing how T1AM and SG‐2 alter cellular metabolism (Fig. 11).

## Conclusions

These data indicate that both SG‐2 and T1AM have strong potential as anticancer treatments due to their effectiveness in the micromolar range. This compares favorably to other metabolic‐based drugs used as anticancer treatments (i.e., metformin), effective in the mm range 26. How SG‐2 and T1AM are specifically impacting cell viability and growth in cancer cells is not entirely clear. However, the cellular imaging data showing T1AM localization to the mitochondria, suggests that such a stress response or membrane perturbations may play a role, reinforcing the metabolic disruption of cancer cell function. The effects of SG‐2 on cancer cell growth rate appear to persist up to a week after cessation of treatment. Uptake of T1AM into both cancer and normal cells is rapid and extensive. Localization to the mitochondria suggests that it is the site of action of T1AM when internalized. The uptake kinetics of T1AM suggests a facilitated diffusion mechanism and that this receptor or channel that mediates uptake is present across multiple cell types. Incubation of cells with SG‐2 inhibited T1AM uptake, suggesting that their uptake is linked through the same mechanism and are likely recognized similarly by cells. The gene expression study suggests that mechanism of action for T1AM and its analog maybe through sirtuin‐mediated pathways particularly at the mitochondrial level (Fig. 11). Considering that the time scale for uptake of T1AM is much faster (in min) vs. its gene activations that requires longer time (in h) to reach for maximum effectiveness of drug action. The longer required time for T1AM action is through gene activation of sirtuins and downstream post‐translational modification processes to support further metabolic effects of T1AM in cancer cells. This study provides ample basis for the use of SG‐2 and T1AM to move forward into *in vivo* study models of cancer to determine efficacy.

## Author contributions

FAP came up with the conceptual ideas. FAP, MR, and LG designed experiments. MR and FAP participated in data collection and analysis. GC made FL‐T1AM reagent. All authors participated in writing and approving the manuscript.

## Supporting information


**Table S1.** Primer sequences used for RT‐PCR analysis.
