# Supplementary material for: Uptake of 3‐iodothyronamine hormone analogs inhibits the growth and viability of cancer cells
Source: FEBS Open Bio. 2017 Mar 6;7(4):587–601. doi: 10.1002/2211-5463.12205 (PMC5377400; doi:10.1002/2211-5463.12205)
Supplement: Supplementary file 1 — Table S1. Primer sequences used for RT‐PCR analysis. [file FEB4-7-587-s001.pdf]

Supplementary Table 1.

Gene	Forward	Reverse
**BCL-2**	GAGTGCTGAAGATTGATG	TCCTCTGTGATGTTGTATT
**G6PD**	CAAGATGATGACCAAGAAG	TTGTATCTGTTGCCGTAG
**GDH**	TGGAGGAGTGACAGTATC	AGCAAGTGGTAGTTAGAATC
**HiF1a**	CCGAGGAAGAACTATGAA	GTTGGTTACTGTTGGTATC
**LDHa**	GCTTGGAAGATAAGTGGTT	CATCAGGTAACGGAATCG
**NF-KB**	AATCATCCACCTTCATTCT	CACATCTTCCTGCTTAGT
**PDHa**	TGGTGAACAGCAATCTTG	AGCATCCTCAATCTCCTT
**p53**	TTACAATCAGCCACATTC	GCCTTGAAGTTAGAGAAA
**Sirt1**	TAGTTCTTGTGGCAGTAA	CATCAGGCTCATCTTCTA
**Sirt4**	CAACCTGCGTTCAATGTG	ACCTTGTCAGGGTTCACT
**Sirt5**	GAAGTCTGTATTATATTGATGTCT	TCTGTAGTTCTAATGCTTGA
**Sirt6**	AGGGACAAACTGGCAGAG	TGTGTCTCGGACGTACTG
