# Supplementary material for: Uptake of 3‐iodothyronamine hormone analogs inhibits the growth and viability of cancer cells
Source: FEBS Open Bio. 2017 Mar 6;7(4):587–601. doi: 10.1002/2211-5463.12205 (PMC5377400; doi:10.1002/2211-5463.12205)
Supplement: Supplementary file 2 [file FEB4-7-587-s002.docx]

**Table S1.** Primer sequences used for RT-PCR analysis.
